# Regional Variation in Dengue Virus Serotypes in Sri Lanka and Its Clinical and Epidemiological Relevance

**DOI:** 10.3390/diagnostics11112084

**Published:** 2021-11-10

**Authors:** Tibutius T. P. Jayadas, Thirunavukarasu Kumanan, Laksiri Gomes, Chandima Jeewandara, Gathsaurie N. Malavige, Diyanath Ranasinghe, Ramesh S. Jadi, Ranjan Ramasamy, Sinnathamby N. Surendran

**Affiliations:** 1 Department of Zoology, University of Jaffna, Jaffna 40000, Sri Lanka; tibutius@univ.jfn.ac.lk; 2 Department of Medicine, University of Jaffna, Jaffna 40000, Sri Lanka; tkumanan@univ.jfn.ac.lk; 3 Centre for Dengue Research, Department of Immunology and Molecular Medicine, University of Sri Jayewardenepura, Nugegoda 10250, Sri Lanka; laksiri79@gmail.com (L.G.); jeewandara@sjp.ac.lk (C.J.); neelika@sjp.ac.lk (G.N.M.); diyanath91@gmail.com (D.R.); 4 Department of Microbiology and Immunology, University of North Carolina School of Medicine, Chapel Hill, NC 27599-7290, USA; ramesh_jadi@med.unc.edu

**Keywords:** clinical manifestations of dengue, Colombo district, dengue diagnosis, dengue epidemiology, dengue virus serotypes, dengue virus genotypes, Jaffna district, salinity tolerant *Aedes* dengue vectors, Sri Lanka

## Abstract

Dengue is a significant health concern in Sri Lanka, but diagnosis of the infecting dengue virus (DENV) serotype has hitherto been largely restricted to the Colombo district in the western province. Salinity tolerant *Aedes* vectors are present in the island’s northern Jaffna peninsula, which is undergoing rapid groundwater salinization. Virus serotypes were determined by RT-qPCR in 107 and 112 patients diagnosed by NS1 antigen positivity from the Jaffna district in 2018 and 2019, respectively, and related to clinical characteristics. DENV1 and DENV2 were the most common serotypes in both years. Infections with multiple serotypes were not detected. DENV1 was significantly more prevalent in 2019 than 2018, while DENV3 was significantly more prevalent in 2018 than 2019 among the Jaffna patients. Limited genomic sequencing identified DENV1 genotype-I and DENV3 genotype-I in Jaffna patients in 2018. Dengue was more prevalent in working age persons and males among the serotyped Jaffna patients. DENV1 and DENV2 were the predominant serotypes in 2019 in the Colombo district. However, DENV1 and DENV3 were significantly more prevalent in Colombo compared with Jaffna in 2019. The differences in the prevalence of DENV1 and DENV3 between the Jaffna and Colombo districts in 2019 have implications for dengue epidemiology and vaccination. Salinity-tolerant *Aedes* vector strains, widespread in the Jaffna peninsula, may have contributed to differences in serotype prevalence compared with the Colombo district in 2019. Significant associations were not identified between virus serotypes and clinical characteristics among Jaffna patients.

## 1. Introduction

**Figure 1:**
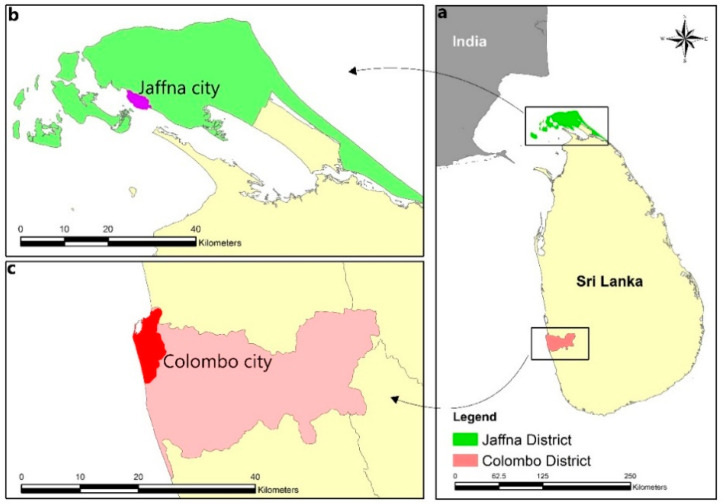
The map shows the (**a**) location of Sri Lanka in relation to India, (**b**) Jaffna city (purple colour) and Jaffna district in the Jaffna peninsula and (**c**) Colombo city (red colour) in the Colombo district.

Dengue fever (DF) is caused by infection with the dengue virus (DENV), which possesses a single, positive RNA strand genome and belongs to the family *Flaviviridae* [1,2,3,4]. Four virus serotypes (DENV1-4) are widely recognized, with each serotype showing limited genomic variations that give rise to several genotypes within each serotype [1,2,3,4]. Dengue is of increasing health concern in tropical and subtropical regions, where the main mosquito vectors *Aedes aegypti* and *Ae. albopictus* are prevalent [1]. Dengue has been documented in Sri Lanka since the beginning of the 20th century, and now causes yearly epidemics [5,6,7,8]. Identification of DENV serotypes has largely been confined to the Colombo district, where the nation’s largest city of Colombo is located (Figure 1), although dengue is now prevalent throughout the island of Sri Lanka [5,6,7,8].

The Jaffna administrative district constitutes most of the northern Jaffna peninsula in Sri Lanka (Figure 1) and is located in the dry zone of the country that receives much of its rainfall during the Northeast monsoon between October and December. The Jaffna district has a land area of 1100 km^2^. Jaffna city is the most populous urban centre and the administrative capital of the Jaffna district with an overall population density of 3048 persons/km^2^. Dengue incidence is seasonal with peak transmission occurring immediately after the onset of monsoon rains in the mainland of Sri Lanka [8] and the Jaffna district [7,8]. As the Jaffna district was relatively isolated from 1983 to 2009 due to a civil war, dengue incidence was recorded in Jaffna only after 2009 [9]. In 2017, 2018 and 2019, respectively, 186,101, 51,659 and 105,049 cases of dengue were reported in the whole of Sri Lanka, and 6075, 4158 and 8261 cases in the Jaffna district [10].

The Jaffna peninsula has a limestone geology and is undergoing rapid salinization of its groundwater aquifers as a result of the unsustainable withdrawal of groundwater for domestic and agricultural purposes [7,11,12], and sea water intrusion due to rising sea levels caused by global warming [13]. As a consequence, *Aedes aegypti* and *Ae. albopictus* in the Jaffna peninsula are more tolerant of salinity than in mainland Sri Lanka [11,14,15]. The two *Aedes* vectors are able to develop in brackish water microhabitats of up to 15 ppt (parts per thousand or gL^−1^) salt in beach litter, and up to 9 ppt salt in domestic wells and surface drains in coastal areas of the Jaffna peninsula [14,15,16,17,18,19]. Salinity-tolerant *Aedes* vectors have since been detected in other countries [20,21,22,23], with worldwide implications for the epidemiology and control of arboviral diseases [24,25,26]. The greater salinity tolerance of *Ae. aegypti* in the Jaffna peninsula is associated with changes in larval structure and physiology, as well as altered insecticide resistance [14,16,27,28]. However, salinity-tolerant *Ae. aegypti* and *Ae. albopictus* in the Jaffna peninsula can be infected with DENV and demonstrate transovarial or vertical transmission of DENV, and such properties impede dengue control because only fresh water *Aedes* habitats are presently targeted for larval source reduction [1,2,7,8].

Because of the paucity of information on infection with DENV serotypes, and the possible influence of biological changes associated with salinity tolerance in *Aedes* vectors in the Jaffna peninsula on the transmission of different DENV serotypes, we determined DENV serotypes and dengue-associated clinical parameters in 2018 and 2019 among patients from the Jaffna district. Findings in Jaffna were compared with DENV serotypes identified in patients from the Colombo district in 2019.

## 2. Materials and Methods

### 2.1. Collection of Clinical Data

A cross-sectional study was performed with written informed consent among dengue patients at the Jaffna Teaching Hospital in the city of Jaffna during the two years from January 2018 to December 2019. Patients suspected to have dengue from the Jaffna district are referred to this hospital for diagnosis and treatment. Dengue is a notifiable disease in Sri Lanka. DF and dengue hemorrhagic fever (DHF) are always considered in the differential diagnosis of patients presenting with acute onset of fever and clinical manifestations of headache, especially retro-orbital pain, myalgia /arthralgia, rash (diffuse, erythematous, macular) and hemorrhage. A full blood count is performed to investigate leukopenia, thrombocytopenia and hematocrit (HCT). Fever with at least two of the clinical manifestations and thrombocytopenia are considered sufficient for diagnosing dengue in Sri Lanka [29,30]. Patients with the evidence of vascular leakage, e.g., pleural effusion and ascites, as ascertained by ultrasound scanning or a rise in the HCT of ≥20%, are classified as having DHF [29,30]. Daily clinical examination findings and the results of pertinent laboratory tests were recorded for each patient. Two hundred and fifty-six such patients aged ≥ 14 y and confirmed to have dengue by the NS1 antigen dip stick test (RapiGEN BIOCREDIT, Heungan-daero, Gyeonggi-do, South Korea) were studied in Jaffna over 2018 and 2019. NS1 antigen-positive dengue patients originating from the Colombo district and admitted to the National Institute of Infectious Disease, Colombo, during 2019 were also investigated for DENV serotypes. Peripheral venous blood, 2–3 mL, was collected from the patients and the separated serum stored at −80 °C.

### 2.2. Dengue Virus Serotyping

DENV serotyping was performed by the widely used United States Centers for Disease Control and Prevention RT-qPCR assay, as previously described [31,32]. Viral RNA from NS1 positive serum samples was extracted with the QIAamp^®^ Viral RNA Mini Kit (Qiagen, Hilden, Germany) and transcribed to cDNA using the high-capacity cDNA reverse transcription kit (Applied Biosystems, Foster City, CA, USA) according to the manufacturer’s protocol. Oligonucleotide primers and labeled probes specific for DENV 1-4 serotypes (Life Technologies, San Francisco, CA, USA) based on published sequences [31,32] were used. The assays were performed with the TaqMan^®^ Multiplex Master Mix (Applied Biosystems, Foster City, CA, USA). The reaction mix of 20 μL contained 1 × TaqMan multiplex master mix (containing Mustang Purple dye), 900 nM of each primer, 250 nM of each probe, 2 μL of cDNA and PCR grade water. Following initial denaturation for 20 s at 95 °C, the reaction was carried out for 40 cycles of 3 s at 95 °C and 30 s at 60 °C on Applied Biosystems—7500 Real Time PCR system.

### 2.3. Dengue Virus Genotyping

One RNA sample each from DENV1 and DENV3 isolated in 2018 from Jaffna patients as described in Section 2.2 was successfully genotyped based on nucleotide sequence variation in the envelope (*E*) and capsid-premembrane (*CprM*) genes. The universal reverse primer (CTRATYTCCATSCCRTACCAGC) was used to generate cDNA with reverse transcriptase (SuperScript™ IV, Thermo Fischer Scientific, Waltham, MA, USA), according to the manufacturer’s protocol. PCR products were generated with primer pairs (forward-GAGACGCAGATCTGCAGGC; reverse-CTRATYTCCATSCCRTACCAGC) using Q5^®^ High-Fidelity DNA Polymerase (New England Biolabs, Ipswich, MA, USA). The PCR conditions were 50 °C for 25 min; initial denaturation 95 °C for 2 min; followed by 35 cycles of 95 °C for 15 s, 57 °C for 30 s, and 63 °C for 2 min and then a final extension at 63 °C for 5 min. PCR products (~2.4 kb) were gel purified and DNA sequenced at Eurofins Genomics, Louisville, KY, USA. The DNA sequences were used to predict genotypes with the Virus Pathogen Database and Analysis Resource (ViPR) computational flavivirus genotyping pipeline version 1.3.0 [33]. A maximum likelihood phylogenetic tree was constructed using Randomized Axelerated Maximum Likelihood (RAxML; version 7.2.6) and the general time reversible (GTR) model with 1000 bootstrap iterations [34].

### 2.4. Statistical Methods

Statistical analysis was done with Prism Graphpad 9.0 (GraphPad Software, San Diego, CA, USA). Pearson’s chi-square test with Yate’s correction was used to compare the numbers of different serotypes in the districts of Colombo and Jaffna in 2019 and within the district of Jaffna for the years 2018 and 2019, with post hoc analysis performed on residuals of Pearson’s chi-square with the Bonferroni correction for pair-wise comparisons. Chi-square tests were also used to analyze the relationships between clinical characteristics of patients infected with different DENV serotypes, as well as the frequencies of dengue infections and different DENV serotypes with gender and the three age groups of Jaffna patients categorized into 14–20 y (school age), 21–60 y (working age) and ≥61 y (senior citizens and retirees). The Kruskal-Wallis one-way analysis of variance test was employed to compare clinical laboratory test results in patients infected with different DENV serotypes. Spearman’s correlation test was performed to correlate laboratory findings with age in all serotyped patients. The Mann–Whitney U test was used to compare laboratory findings in patients with DF and DHF. Statistical significance was set at *p* ≤ 0.05.

## 3. Results

Findings on the dengue serotypes identified in Jaffna patients in relation to the year of infection, age group and gender are shown in Table 1.

### 3.1. DENV Serotypes in 2018 and 2019

DENV serotyping was successful in 107 and 112 patients in 2018 and 2019, respectively (Table 1), out of the total of 256 NS1-reactive Jaffna patients over the two-year study. All four serotypes were detected in the patients in 2018 and 2019, with a preponderance of DENV1 and DENV2 over DENV3 and DENV4 (Table 1). Co-infection with two or more serotypes was not observed. The Pearson’s chi-square test showed significant differences in the frequency of the four serotypes between 2018 and 2019 (χ^2^ = 26.616; *p* < 0.001) among the Jaffna patients. Post hoc pair-wise comparisons demonstrated a significantly greater frequency of DENV1 in 2019 compared with 2018 (*p* < 0.001) and DENV3 in 2018 compared with 2019 (*p* = 0.005) among the Jaffna patients.

**Figure 2:**
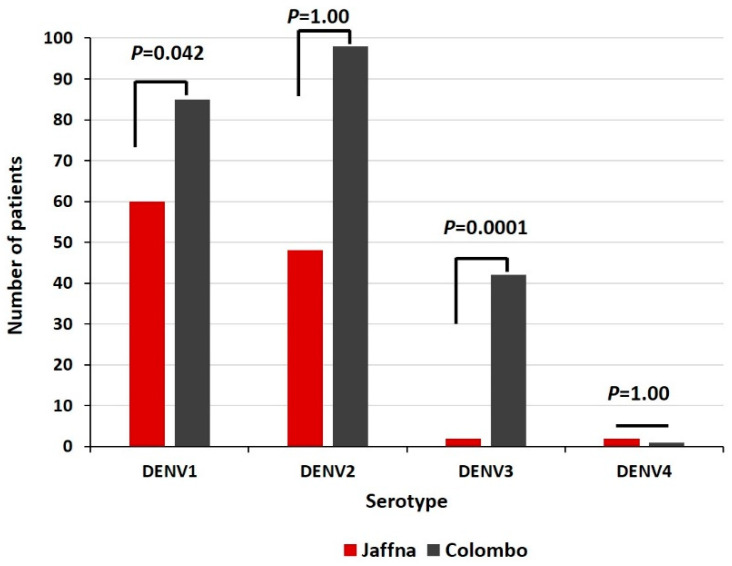
Numbers of DENV serotypes in 2019 among Jaffna and Colombo patients.

Two hundred and twenty-six patient sera were successfully serotyped in 2019 in the Colombo district, of which 85, 98, 42 and 1 were identified as being infected with DENV1, DENV2, DENV3 and DENV4, respectively. Infections with multiple serotypes were not detected. Although DENV 1 and DENV2 were the more frequent serotypes identified in both districts, there were significant differences between the Jaffna and Colombo patients in the frequencies of the four serotypes collectively (χ^2^ = 22.21; *p* < 0.0001), and individually, with the Colombo patients demonstrating increased frequencies of DENV1 (*p* = 0.042) and DENV3 (*p* < 0.0001) in 2019 in pair-wise comparisons (Figure 2).

### 3.2. Age and Gender in Relation to Individual Serotypes and Total Number of Serotyped Dengue Cases in Jaffna

The Pearson’s chi-square test did not demonstrate significant relationships between different DENV serotypes and gender (χ^2^ = 7.138; *p* = 0.068) or different serotypes and the three age groups (χ^2^ = 2.377; *p* = 0.881) in Jaffna. However, the chi-square test revealed a significant difference between the frequencies of the total number of dengue cases in the three age groups (χ^2^ = 143.17; *p* < 0.0001), with the highest frequency of cases occurring in the working age group (20–60 y). The frequency of dengue cases was significantly higher among male patients in Jaffna (χ^2^ = 9.94; *p* = 0.0016).

### 3.3. DENV Genotypes Identified in Jaffna in 2018

Nucleotide sequences covering overlapping genomic regions of the *CprM* and *E* genes of DENV1 and DENV3 were obtained from single isolates of DENV1 and DENV3 from Jaffna patients in 2018, and deposited in GenBank with the respective accession numbers MZ676072 and MZ677048. A maximum likelihood phylogenetic tree constructed from the two sequences and standard reference genotype sequences (Figure S1) established that two isolates belonged to DENV1 genotype 1 (DENV1/I) and DENV3 genotype 1 (DENV3/I). The two Jaffna DENV sequences also showed ≥99% sequence identity by National Centre for Biological Information BLASTn analysis with the Sri Lankan DENV sequences JN054255 and MN083246 in GenBank that had been obtained from mainland Sri Lanka viral isolates in 2010 and 2017, respectively.

### 3.4. Clinical Manifestations and Laboratory Findings in Relation to DENV Serotypes in Jaffna

Infection with the four serotypes resulted in similar common clinical manifestations in Jaffna, with no statistically significant differences between infecting serotypes (Table S1).

Clinical laboratory findings in the Jaffna patients in relation to DENV serotypes are shown in Table S2. Leukocytopenia, neutropenia, lymphocytopenia, thrombocytopenia and elevated levels of the liver enzymes alanine transaminase (ALT) and aspartate transaminase (AST) were found in patients with no statistically significant difference between the four serotypes (Table S2). Out of the total 219 serotyped dengue patients, 206 and 13 were identified with DF and DHF, respectively. Neutrophil counts, HCT, ALT and AST were elevated, while platelet counts were decreased in DHF compared with DF (Table S3). A positive correlation was also found between age and elevated ALT for infections with DENV1 (r = 0.274; *p* = 0.016) and DENV3 (r = 0.574; *p* = 0.018) as well as elevated AST with DENV3 (r = 0.647; *p* = 0.05).

## 4. Discussion

Serotyping was successful only in 219 of the 256 NS1 antigen-positive patient sera from Jaffna. The slower clearance of NS1 protein compared with viral RNA from blood during a resolving dengue infection is a likely cause of negativity in the RT-qPCR assay in a proportion of NS1-antigen positive patients [35,36,37,38].

Changing epidemiology in terms of dengue incidence and disease severity has been associated with antigenically distinct DENV serotypes [39,40]. DENV1 was the predominant circulating serotype in Colombo until the emergence of DENV2 as the predominant serotype in mid-2016 [5]. The sudden emergence of DENV2 in Colombo, after a complete absence of this serotype for eight years, led to the largest-ever dengue epidemic in Sri Lanka in 2017 [41]. A study in Singapore, based on 14-years of data from 2004 to 2016, showed that the predominant serotypes were DENV1 and DENV2, and that oscillations in the prevalence of the two serotypes provided an early indication of epidemics, which may have resulted from the waning of serotype-specific immunity with time [42]. This may also contribute to the recurrent pattern of dengue epidemics in Sri Lanka.

Sequence analysis of a 2018 DENV1 isolate from the Jaffna district revealed it to be genotype DENV1/I, which was first identified in 2009 in Sri Lanka [43]. DENV1/III circulated during 1983 to 1984 and DENV1/II from 1984 until 2004 in Sri Lanka. Since its appearance in 2009, DENV1/I has replaced all the other DENV1 genotypes in the country [6,43,44]. DENV3/I, detected in the present report in Jaffna in 2018, first appeared in Sri Lanka during the major 2017 dengue epidemic, replacing DENV3/III that had been circulating during the 1980s and 1990s. The role of genotype changes, in addition to serotype changes, in the context of immune responses, recurrence of dengue epidemics and different clinical manifestations, therefore merits detailed investigation.

The age group 21–60 y was most infected with DENV among the serotyped Jaffna patients, and this mainly represented the working adult population who are likely to be exposed to day time biting of infected *Aedes* vector mosquitoes outdoors and at work places. Similar findings have been made in other countries [45,46]. Significant differences between infection with different serotypes and age groups were not found in Jaffna, but this may have been influenced by the smaller number of serotyped patients studied.

Greater exposure to daytime biting by *Aedes* vectors outdoors and during working hours may also be responsible for the higher number of dengue cases among males in Jaffna. Similar findings have been made in other studies [45,47]. Significant differences between infection with different serotypes and gender were not found in Jaffna, unlike in Taiwan [45], but this may also be due to the smaller numbers of serotyped patients studied in Jaffna.

Differences between the four DENV serotypes in clinical symptoms and laboratory findings have also been reported in studies from other countries [45,48,49]. This may be related to variations in infectivity and pathogenicity between the DENV serotypes and also host genetic characteristics. The lack of demonstrable differences in Jaffna may have been influenced by the smaller numbers of serotyped patients available for study, in particular those with DENV3 and DENV4. Elevated ALT and AST levels are a common indication of the liver involvement in dengue, and unlike other types of viral hepatitis, the level of AST is generally higher than ALT in dengue [50,51]. The positive correlation between increased liver enzyme levels and age in Jaffna patients may be due to other morbidities that increase in prevalence with age that may aggravate liver involvement in dengue [45,50,52]. The laboratory findings in Jaffna patients also illustrate greater liver damage and vascular leakage in DHF compared with DF.

*Aedes aegytpi* and *Ae. albopictus* are present in urban and semiurban areas of the Jaffna district, including the city of Jaffna [7]. It is pertinent that DENV2 was identified in *Ae. aegypti* larvae collected from ovitraps in Jaffna city in 2019 [7]. Different DENV serotypes have been reported to variably infect *Ae. aegypti* and *Ae. albopictus* [53]. Furthermore, relative infectivity of DENV to *Ae. aegypti* has been shown to vary between different strains of *Ae. aegypti* [54]. *Aedes aegypti* and *Ae. albopictus* in the Jaffna peninsula have been demonstrated to possess greater salinity tolerance than the corresponding vectors from mainland Sri Lanka [14,15,16,17], and this property has been associated with specific structural and physiological changes in *Ae. aegypti* [14,27,28]. The possibility that salinity-tolerant *Aedes* vectors in the Jaffna district have different vectorial capacities for the four DENV serotypes compared with fresh water vectors in the Colombo district is therefore a possibility that requires further investigation.

There were significant differences in the numbers of infections with DENV1 and DENV3 between 2018 and 2019 within the serotyped patients in the Jaffna district. DENV1 and DENV3 serotypes were significantly more frequent in the Colombo district than in the Jaffna district among the patients serotyped in 2019. Serotype data for 2018 in the Colombo district and for other years in the Jaffna district were not available for comparison. These serotype variations have to be viewed in the context of Sri Lanka being a relatively small island with a land area of 65,268 km^2^, and an approximate distance of 265 km between the Jaffna and Colombo districts. The Jaffna peninsula has extensive transport links with the Colombo district that facilitate significant movement of people between the two areas for work, government administration and social purposes. Such population movement can be expected to rapidly homogenize DENV strains between the Jaffna and Colombo districts, because dengue transmission is closely related to the movement of people [55,56,57]. Our findings however show a significant variation in the frequencies of DENV1 and DENV3 serotypes in the Jaffna and Colombo districts in 2019. It is possible to hypothesize that multiple, and possibly interacting, factors may have contributed to the observed variation: (i) local transmission being the predominant cause of dengue infections in the Jaffna district, (ii) serotype-dependent variation in the transmission of DENV to biologically different and geographically separated *Aedes* vectors in the Jaffna and Colombo districts as discussed above, (iii) dengue incidence peaking after both the Southwest monsoon from May to July and Northeast monsoon from October to December in Colombo, and mainly after the Northeast monsoon from October to December in Jaffna, and (iv) the relatively small numbers of dengue cases that were available for this study, that was also performed over a short time period. Additional investigations involving continuing observations over several years and larger numbers of dengue patients are needed to establish to what extent each of these possibilities may have contributed to the variations in the frequency of different DENV serotypes between Jaffna and Colombo districts in 2019. Studies on the variations in the frequency of the four DENV serotypes in different areas of the country will also be useful for understanding changes in serotype-specific immunity and the relevance to antibody-dependent enhancement of infection and immunopathology [58,59,60]. This can facilitate the development and use of vaccines for dengue that can vary in efficacy against different serotypes and produce antibody-dependent enhancement of DENV infectivity [61,62], and also in ascertaining the relevance of localized foci of specific DENV serotypes for initiating dengue epidemics in surrounding areas. Determination of serotypes in regions with varying ecology and geography within the island over several consecutive years will therefore be helpful in more firmly establishing regional and temporal variations in serotype heterogeneity, and be useful for dengue control efforts and the clinical management of patients.

## Figures and Tables

**Table 1:** DENV serotypes identified in the Jaffna district in relation to year, patient age group and gender.

Category	DENV Serotype				Total
	DENV1 (%)	DENV2 (%)	DENV3 (%)	DENV4 (%)	
**Year**					
2018	26 (24)	60 (56)	15 (14)	6 (6)	107
2019	60 (53)	48 (43)	2 (2)	2 (2)	112
Total	86	108	17	8	219
**Age group**					
14–20	29 (41)	34 (48)	5 (7)	3 (4)	71
21–60	53 (40)	64 (48)	11 (8)	5 (4)	133
≥61	4 (27)	10 (67)	1 (6)	0 (0)	15
**Gender**					
Male	45 (36)	64 (51)	9 (7)	8 (6)	126
Female	41 (44)	44 (47)	8 (9)	0 (0)	93
Total	86	108	17	8	219

The number of patients infected with each serotype and the corresponding percentage of the respective totals in parentheses are shown.

## Data Availability

All datasets supporting the conclusions of this article are included within the article.

## Supplementary Materials

The following re available online at https://www.mdpi.com/article/10.3390/diagnostics11112084/s1, Figure S1: Genotype tree of Jaffna DENV1-I and DENV3-I sequences. Table S1: Clinical manifestations in relation to DENV serotypes. Table S2: Laboratory findings in relation to DENV serotypes. Table S3: Laboratory findings commonly used in dengue diagnosis and their association with dengue fever (DF) and dengue hemorrhagic fever (DHF) in serotyped Jaffna patients.
