# Supplementary material for: Regional Variation in Dengue Virus Serotypes in Sri Lanka and Its Clinical and Epidemiological Relevance
Source: Diagnostics (Basel). 2021 Nov 10;11(11):2084. doi: 10.3390/diagnostics11112084 (PMC8618005; doi:10.3390/diagnostics11112084)
Supplement: Supplementary file 1 [file diagnostics-11-02084-s001.zip › diagnostics-1435590-supplementary.pdf]

Figure S1: Genotype tree of Jaffna DENV1-I and DENV3-I sequences.

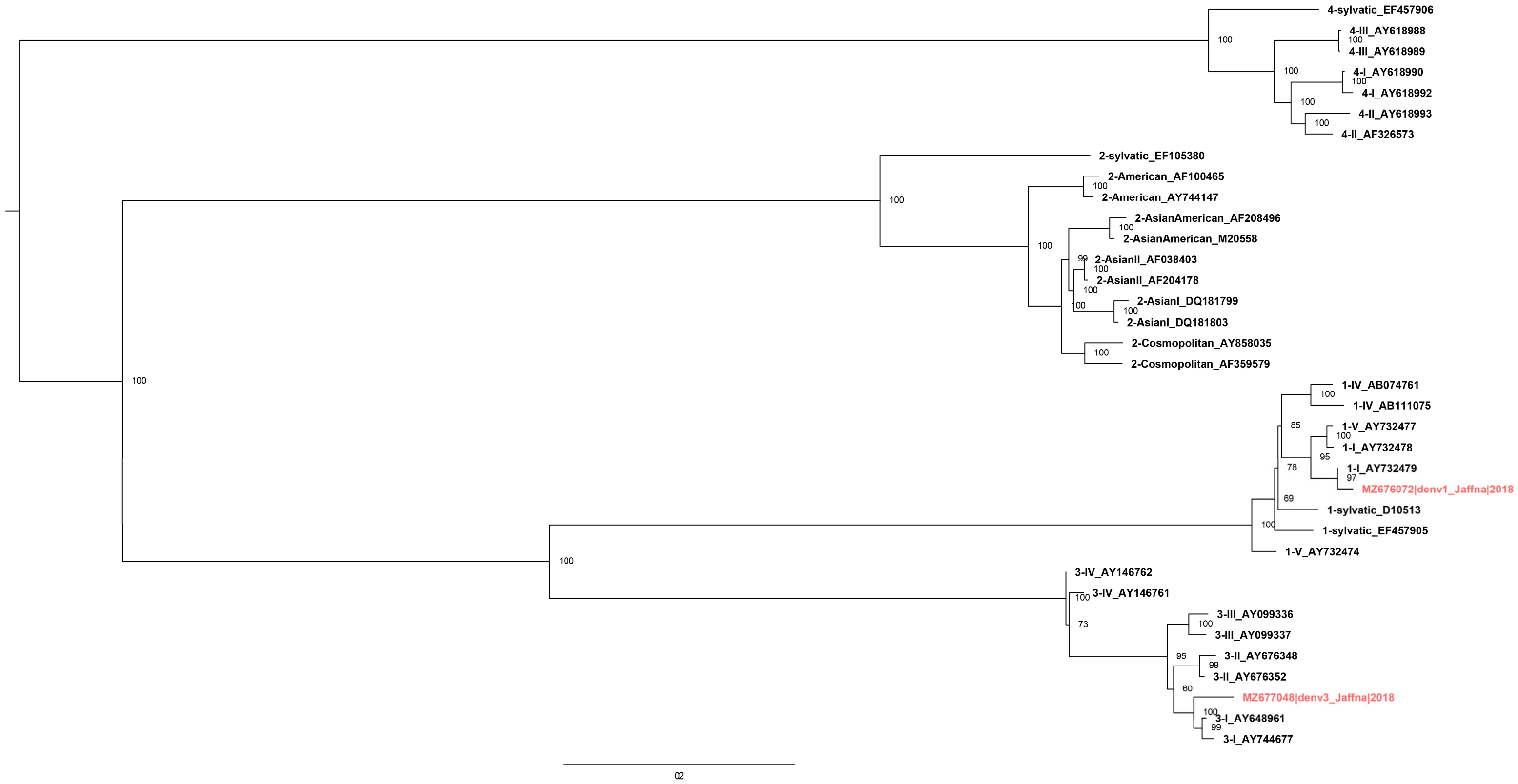

Legend to Figure: Midpoint rooted maximum likelihood phylogenetic tree of DENV1and DENV3 sequences from Jaffna (highlighted in red text) with the reference genotyping sequences recommended in the ViPR computational Flavivirus genotyping programme version 1.3.0 (https://github.com/VirusBRC/Flavivirus_Genotyping) and generated in Randomized Axelerated Maximum Likelihood (RAxML, version 7.2.6) with the general time reversible (GTR model) using 1,000 bootstrap iterations [33,34]. Bootstrap support values are shown at each node.

Table S1: Clinical manifestations in relation to DENV serotypes.

Symptoms	DENV1 N (%)	DENV2 N (%)	DENV3 N (%)	DENV4 N (%)	Total N (%)	*P* Value
Fever	86 (100)	108 (100%)	17 (100%)	8 (100%)	219 (100%)	N/A
Headache	48 (56%)	64 (59%)	11 (65%)	4 (50%)	127 (58%)	0.858
Retroorbital pain	4 (5%)	9 (8%)	3 (18%)	2 (25%)	18 (8%)	0.091
Myalgia	31 (36%)	44 (41%)	6 (35%)	2 (25%)	83 (38%)	0.774
Arthralgia	16 (19%)	21 (19%)	3 (18%)	2 (25%)	42 (19%)	0.973
Backpain	2 (2%)	2 (2%)	1 (69%)	0 (0%)	5 (2%)	0.737
Anorexia	12 (14%)	24 (22%)	4 (24%)	1 (13%)	41 (19%)	0.454
Nausea	37 (43%)	40 (37%)	6 (35%)	2 (25%)	85 (39%)	0.678
Vomiting	23 (27%)	27 (29%)	5 (29%)	1 (13%)	56 (26%)	0.818
Abdominal pain	5 (6%)	11 (10%)	2 (12%)	1 (13%)	19 (9%)	0.67

Legend to Table S1. Probability values are the outcome of the chi-square tests. N=number of patients with respective proportions shown in parentheses.

Table S2: Laboratory findings in relation to DENV serotypes.

Laboratory Findings		DENV1 N (%)	DENV2 N (%)	DENV3 N (%)	DENV4 N (%)	Total
Leucocytes (<5 x 10^9/L)		76 (88%)	90 (83%)	15 (88%)	7 (88%)	188 (86%)
Neutrophils (<2 x 10^9/L		55 (64%)	71 (66%)	15 (88%)	6 (75%)	147 (67%)
Lymphocytes(<1 x 10^9/L)		35 (41%)	50 (46%)	8 (47%)	4 (50%)	97 (44%)
Hb (>16mg/dL)		7 (8%)	6 (6%)	2 (12%)	2 (25%)	17 (8%)
HCT	10% increase	43 (50%)	38 (35%)	8 (47%)	5 (63%)	94 (43%)
	20% increase	9 (10%)	7 (6%)	0	0	16 (7%)
Platelets (<100x10^9/L)		63 (73%)	88 (81%)	11 (65%)	6 (75%)	168 (77%)
ALT (>63U/L)		34 (40%)	45 (42%)	7 (41%)	4 (50%)	90 (41%)
AST (>37U/L)		62 (72%)	90 (83%)	15 (88%)	5 (63%)	172 (79%)

Legend to Table S2. N = number of serotyped Jaffna patients with respective proportions shown in parentheses, Hb = Hemoglobin, HCT = Hematocrit AST = aspartate transaminase, ALT = alanine transaminase.

Table S3: Laboratory findings commonly used in dengue diagnosis and their association with dengue fever (DF) and dengue haemorrhagic fever (DHF) in the serotyped Jaffna patients.

**Laboratory Parameters**	**DF**	**DHF**	***P* value**
Number of patients	206	13	
Leucocytes (x 10^9/L)	3.6 ± 1.9	4.2 ± 2.1	0.32
Neutrophils (x 10^9/L)	1.8 ± 1.2	2.5 ± 1.4	0.05
Lymphocytes (x 10^9/L)	1.2 ± 0.7	1.2 ± 0.6	0.99
Hb (mg/dL)	13.5 ± 1.7	14.8 ± 2.7	0.0972
HCT (%)	9.4 ± 5.3	24 ± 6.5	<0.0001
Platelets (x 10^9/L)	73.8 ± 46.9	27.1 ± 18.3	<0.0001
ALT (U/L)	91.3 ± 113.8	288.5 ± 431.9	0.003
AST(U/L)	120 ± 166	394.7 ± 576.3	0.009

Legend to Table S3: Probability values are the outcome of the Mann–Whitney U test (N = number of patients, Hb = Hemoglobin, HCT = Hematocrit, AST = aspartate transaminase, ALT = alanine transaminase).
